# Transcatheter aortic valve replacement for left ventricular assist device-related aortic regurgitation and prohibitive surgical risk: a case report

**DOI:** 10.1093/ehjcr/ytaf027

**Published:** 2025-01-23

**Authors:** Andrés F Barragán-Amado, María Manzur Barbur, Diego Holguín, Carlos Obando, María J Rodríguez-González

**Affiliations:** Division of Cardiology, LaCardio-Fundación Cardioinfantil, Calle 163a # 13b-60, Bogotá, Colombia; Division of Heart Failure and Cardiac Transplantation, LaCardio-Fundación Cardioinfantil, Calle 163a # 13b-60, Bogotá, Colombia; Division of Cardiology, LaCardio-Fundación Cardioinfantil, Calle 163a # 13b-60, Bogotá, Colombia; Division of Cardiology, LaCardio-Fundación Cardioinfantil, Calle 163a # 13b-60, Bogotá, Colombia; Division of Cardiology, LaCardio-Fundación Cardioinfantil, Calle 163a # 13b-60, Bogotá, Colombia; Division of Heart Failure and Cardiac Transplantation, LaCardio-Fundación Cardioinfantil, Calle 163a # 13b-60, Bogotá, Colombia; Division of Cardiovascular Surgery, LaCardio-Fundación Cardioinfantil, Calle 163a # 13b-60, Bogotá, Colombia; Division of Cardiology, LaCardio-Fundación Cardioinfantil, Calle 163a # 13b-60, Bogotá, Colombia; Division of Heart Failure and Cardiac Transplantation, LaCardio-Fundación Cardioinfantil, Calle 163a # 13b-60, Bogotá, Colombia

**Keywords:** Aortic regurgitation, Left ventricular assist device, Heart transplantation, Heart failure, Advanced heart failure, Case report

## Abstract

**Background:**

Aortic regurgitation (AR) is a prevalent complication following left ventricular assist device (LVAD) implantation, which elevates the risk of mortality. Prompt recognition and intervention are crucial to mitigate this risk.

**Case summary:**

A 52-year-old male with severe left ventricular dysfunction secondary to Chagas cardiomyopathy and severe pulmonary hypertension underwent implantation of a HeartMate 3 device as a bridge to transplant candidacy. Post-implantation, he developed progressive AR and a decline in overall health, characterized by right ventricular dysfunction, worsening mitral regurgitation, and elevated pulmonary pressures. Given the very high surgical risk, transcatheter aortic valve replacement was successfully performed, yielding excellent outcomes, including normalization of right ventricular function, reduction in pulmonary pressures, and a decrease in mitral regurgitation.

**Discussion:**

Aortic regurgitation is a progressive complication in patients with continuous flow-LVAD, limits effective blood flow, and can lead to severe outcomes like biventricular failure, high rates of hospitalization, and mortality. Managing AR is challenging, often requiring percutaneous interventions due to high surgical risks. Treatment choices depend on centre expertise and patient specifics.

**Learning points**

- Aortic regurgitation (AR) is common in patients with continuous flow-left ventricular assist device (LVAD). Many theories about the origin of AR.
- Moderate or severe AR occurs in up to 52% of LVAD recipients within the first three years.
- More than mild AR must be communicated to the heart team to increase the follow-up.
- There are pros and cons of surgical or percutaneous management strategies for the native aortic valve.
- It is crucial for healthcare professionals to possess the knowledge and expertise to effectively manage *de novo* AR post-LVAD implant, based on the team’s experience in this field.

## Introduction

Advanced heart failure represents the end-stage of the disease, with high mortality regardless of the use of pharmacological or device therapies.^1^ In this group of patients, heart transplant or long-term mechanical circulatory support devices are the last options which reduce mortality, improve functional class, and quality of life.^1,2^

Left ventricular assist device (LVAD) implant rates are increasing in relation to the low donor pool, so to be aware of complications is essential for the heart team to establish treatments, long-term follow-up, and possible new approaches.^2^ Aortic regurgitation (AR) is one of the possible complications after LVAD implantation, potentially dangerous for the LVAD performance and is associated with high rates of hospitalization and mortality. Currently, with Class 1 recommendation in patients with moderate or severe AR at the moment of LVAD implantation, the bioprosthetic aortic valve replacement should be favoured,^3^ however, there is no guideline recommendation on the management of this pathology in patients after LVAD implantation and recommendations on the type of intervention are based on the experience of different groups, considering that transcatheter aortic valve replacement (TAVR) has initially emerged for the management of severe aortic stenosis, however, it has become a potential treatment for isolated AR with susceptible anatomy and prohibitive surgical risk.^4–6^

## Summary figure

Timeline events with echocardiographic and fluoroscopic evaluation, pre- and post-procedural. Pre-procedural TTE evaluation. (*A*) PLAX colour view demonstrates severe aortic regurgitation and mitral regurgitation with right ventricular dilatation. (*B*) M-mode imaging shows the absence of valve opening, and (*C*) zoom greatest vessels projection on the image reveals the presence of both AR and tricuspid regurgitation. (*D*) demonstrated fluoroscopic images with notable contrast regurgitation, (*E*) shows a pre-implantation position and evaluation, and (*F*) the final procedural image exhibited an adequate valvular implantation without residual AR. (*G*) The post-procedural control TTE revealed PLAX colour view with absence of AR, (*H*) M-mode imaging shows the absence of valve opening, and (*I*) zooming in on the greatest vessels indicated a reduction in tricuspid regurgitation and an absence of colour in the aortic valve with AR correction.

**Figure:**
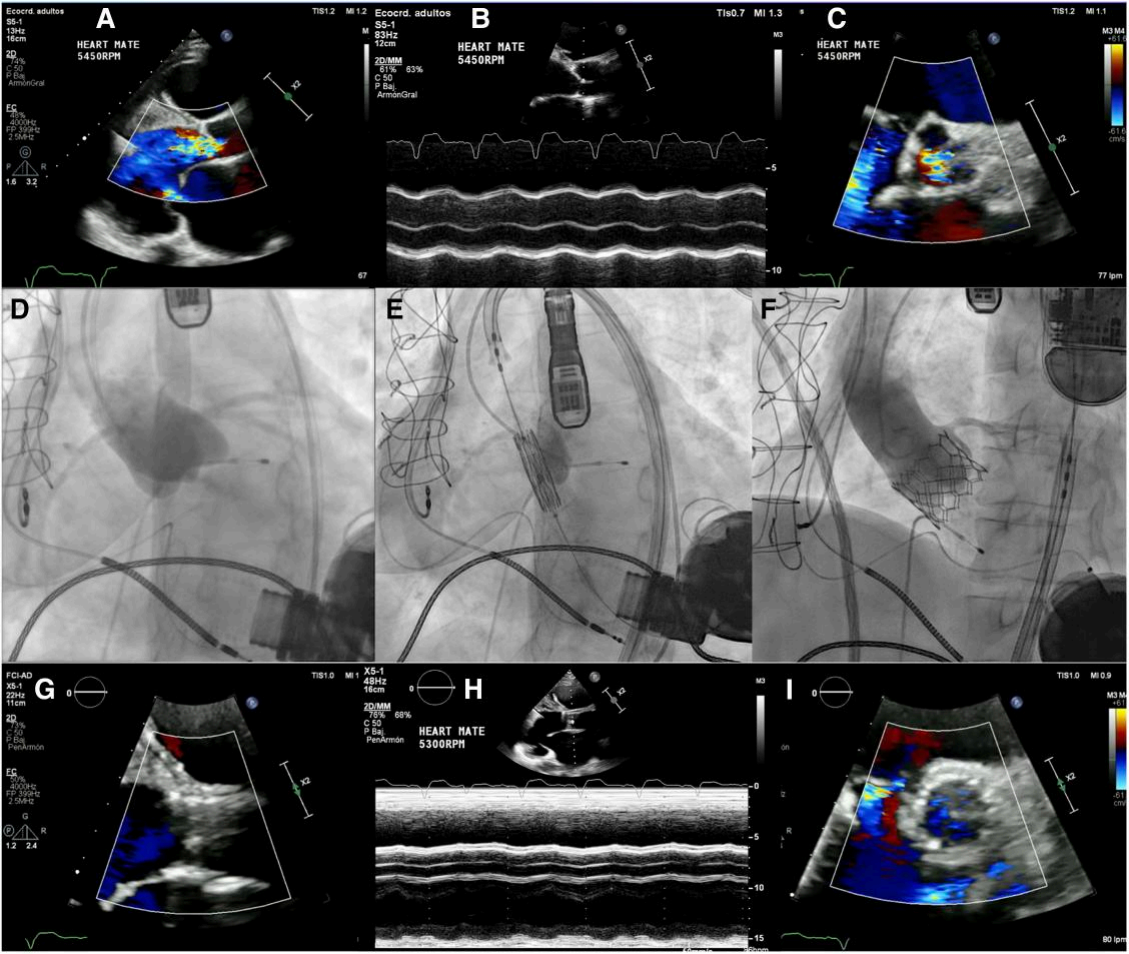


## Case report

Male of 52 years old with 10 years of medical history of advanced heart failure secondary to Chagas cardiomyopathy and severe non-reactive pulmonary hypertension, a LVAD (HeartMate 3) implant was performed in July 2022. However, continuous opening of the aortic valve was not achieved completely after implantation. At one month follow-up, mild AR was demonstrated, without heart failure signs and haemodynamic repercussions, and echocardiographic follow-up was considered. At the 10th month’s follow-up, the patient presented at the heart failure day hospital with a deterioration of functional class up to NYHA III, rales, bilateral pleural effusion, abdominal distention, and bilateral leg oedema. Echocardiographic control showed progression to moderate AR, with a significant increase in left ventricular end-diastolic diameter and severe functional mitral regurgitation, as well as right ventricular repercussion due to the increase in pulmonary pressures. The LVAD did not show any relevant alarm. We initiated ambulatory intravenous loop diuretics administration for 3 days. A structural pathology meeting was convened, considering the high risk of LVAD failure and high risk of redo surgery. The meeting indicated further studies with right catheterization and tomography with TAVR protocol to perform it as an elective intervention.

After a period of two weeks, the patient was admitted to the emergency room with a deterioration in symptoms, severe leg oedema, and signs of pulmonary oedema, indicative of new heart failure decompensation. Conventional laboratory tests did not reveal the presence of anaemia, haemolysis, or significative electrolyte disturbances, the INR was 2.94, and renal function was normal. However, the patient exhibited a significant increase in NT-proBNP, prompting the performance of transoesophageal echocardiography (TTE), which revealed severe left ventricular dilatation (LVEDD 78 mm) and progression to severe AR without thrombosis signs. Additionally, TTE revealed an increase in the severity of mitral regurgitation with a coaptation defect and right ventricular dilatation with moderate systolic dysfunction. M-mode imaging revealed the absence of valve opening, and zooming in on the greatest vessels demonstrated the presence of both AR and tricuspid regurgitation (*Summary figure*, *Panels A–C*; see Supplementary material online, *Videos S1 and S2*). Following a week of treatment with intravenous loop diuretics and optimization of oral vasodilator therapy, the patient underwent right heart catheterization, which revealed severe post-capillary pulmonary hypertension. Additionally, a chest CT scan with protocol for TAVI was performed, and severe dilatation of the left ventricle was observed, indicating an apparent increase compared to the previous study. Subsequent measurements were conducted, which revealed favourable anatomy for percutaneous valve implantation. The patient was presented in institutional heart team meeting with international proctors, considering that the best option for treatment due to the institutional experience in TAVR implantation in the context of AR was this procedure. Fluoroscopy images demonstrate a substantial regurgitation of contrast medium through the aortic valve, accompanied by a markedly dilated left ventricle, and a transcatheter balloon-expandable aortic valve 29 mm was successfully implanted (*Summary figure*, *Panels D–F*), and by transoesophageal echocardiography guidance, a marked reduction in the severity of mitral regurgitation was found, with a significant reduction in sPAP (34 mmHg) and better alignment of the interventricular septum. Following the implantation procedure, the patient was transferred to the intensive care unit for continuous haemodynamic monitoring. During the ensuing 48-h period, the patient did not require any additional mechanical or pharmacological support, and after two weeks of hospitalization, the Doppler colour study on TTE did not show the presence of AR, and the M-mode study showed no valve opening in all cycles (*Summary figure*, *Panels G–I*; see Supplementary material online, *Videos S3–S5*). Right heart catheterization was performed, and normalization of mPAP (11 mmHg) was noted. The patient was discharged with the directive for monthly follow-ups at the specialized heart failure day hospital.

Currently, our patient completes 8 months of active follow-up after TAVR implantation, with significant improvement in functional class, and the TTE follow-up demonstrated a prosthetic aortic valve without opening but without regurgitant jet, reduction of the LV diameters, and severity of mitral regurgitation echocardiogram as well as in sPAP and no LVAD alarms. In the subsequent right catheterization, a reduction in pulmonary pressures was observed, resulting in the patient’s re-inclusion on the transplant list.

## Discussion

Advanced heart failure affects 10% of patients with heart failure, and mortality rates remain high even after the implementation of guideline-directed medical therapies.^1^ Continuous flow (CF)-LVADs, particularly the HeartMate 3, are the primary destination therapy for patients with non-reactive pulmonary hypertension who are not candidates for heart transplantation. Aortic regurgitation is a progressive complication in patients with CF-LVADs.^2^ The prevalence of clinically significant AR varies widely, ranging from 6% in the preliminary reports of the Momentum 3 Trial^3^ to 52% within the first three years in other registries. This prevalence is related to the type of device, with higher rates observed in patients with the HeartMate 2.^4^

The mechanisms contributing to AR include commissural fusion and leaflet tissue deterioration, which occur in the absence of pulsatile flow over the aortic root.^2^ AORTIC regurgitation induces a regurgitant flow loop that limits effective forward flow, leading to organ dysfunction, increased risk of haemolysis and thrombosis, elevated left ventricular diastolic pressures, and eventually progression to biventricular failure, LVAD dysfunction, and high rates of hospitalization and mortality.^5^ Left ventricular assist device patients with at least mild AR have increased filling pressures and reduced pulmonary artery pulsatility index (PAPI). Managing this complication is challenging for the heart team. Initially, increasing the LVAD speed to alleviate heart failure symptoms is an option; however, this adjustment decreases left ventricular pressure and increases the aortic valve-left ventricular gradient, which can exacerbate AR, making it a temporary solution for symptom relief.^6^

Given the prohibitive surgical risk in these patients, as demonstrated in our case, percutaneous devices have emerged as a strong and increasingly utilized therapeutic option in specialized centres. The available options include the use of transcatheter prostheses or occlusion devices.^7,8^ Short-term haemodynamic outcomes have been favourable with both, showing a decrease in left ventricular dilation, improved alignment of the interventricular septum, correction of right ventricular dysfunction, and improvements in parameters such as PAPI.^5–7^ Other types of interventions, such as percutaneous aortic valve closure, have become less common due to increased mortality rates, particularly in LVAD-dependent patients. Sudden power loss, pump thrombosis, or other mechanical failures could result in fatal outcomes in this population.^7,8^

The presence of valvular heart disease, particularly AR, in patients with CF-LVAD support presents unique challenges that are currently managed on a case-by-case basis due to the lack of evidence-based guidelines. The absence of annular calcifications in CF-LVAD patients poses an additional challenge, as it may precipitate device migration during the TAVR procedure.^8^ Therefore, the management of these patients should be multidisciplinary, with simulations based on each patient’s individual characteristics, and performed in highly experienced centres.

In our case, we opted for transcatheter implantation of a balloon-expandable valve due to our centre’s experience with this type of valve in cases of aortic insufficiency. However, the choice of therapy will depend on each centre’s expertise and experience.

## Supplementary Material

ytaf027_Supplementary_Data

## Data Availability

The data underlying this article can be shared on reasonable request to the corresponding author.

## Lead author biography

Dr Andrés Felipe Barragán-Amado is a cardiologist and belongs to the heart failure and heart transplantation group at LaCardio-Fundación Cardioinfantil in Bogotá, Colombia. He is currently doing a fellowship in advanced heart failure and heart transplantation at the Hospital Clinic in Barcelona, Spain. He has special interest in LVADs and acute heart failure.

## Supplementary material

**Consent:** The authors confirm that written consent for submission and publication of this case report including images and associated text has been obtained from the patient in line with COPE guidelines.

**Funding:** None declared.
